# The serum soluble scavenger with 5 domains levels: A novel biomarker for individuals with heart failure

**DOI:** 10.3389/fphys.2023.1140856

**Published:** 2023-04-13

**Authors:** Yulong Ge, Xiaoqiang Liu, Hangwei Chen, Gonghao Li, Xing Xing, Junyi Liu, Chunxia Zhang, Ying Zhuge, Fang Wang

**Affiliations:** ^1^ Department of Cardiology, Shanghai General Hospital, Shanghai Jiao Tong University School of Medicine, Shanghai, China; ^2^ Department of Cardiology, The First People’s Hospital of Lianyungang, Xuzhou Medical University Affiliated Hospital of Lianyungang, Lianyungang, China

**Keywords:** heart failure, SSc5D, biomarker, LVEF, NT-ProBNP

## Abstract

**Background:** We aimed to explore the relationship between the serum Soluble Scavenger with 5 Domains (SSC5D) levels and heart failure (HF).

**Methods and Results:** We retrospectively enrolled 276 patients diagnosed with HF or normal during hospitalization in Shanghai General Hospital between September 2020 and December 2021. Previously published RNA sequencing data were re-analyzed to confirm the expression profile of *SSC5D* in failing and non-failing human and mouse heart tissues. Quantitative real-time polymerase chain reaction assay was used to quantify *Ssc5d* mRNA levels in murine heart tissue after myocardial infarction and transverse aortic constriction surgery. To understand the HF-induced secreted proteins profile, 1,755 secreted proteins were investigated using human dilated cardiomyopathy RNA-seq data, and the results indicated that *SSC5D* levels were significantly elevated in failing hearts compared to the non-failing. Using single-cell RNA sequencing data, we demonstrated that *Ssc5d* is predominantly expressed in cardiac fibroblasts. In a murine model of myocardial infarction or transverse aortic constriction, *Ssc5d* mRNA levels were markedly increased compared with those in the sham group. Similarly, serum SSC5D levels were considerably elevated in the HF group compared with the control group [15,789.35 (10,745.32–23,110.65) pg/mL, 95% CI (16,263.01–19,655.43) vs. 8,938.72 (6,154.97–12,778.81) pg/mL, 95% CI (9,337.50–11,142.93); *p* < 0.0001]. Moreover, serum SSC5D levels were positively correlated with N-terminal pro-B-type natriuretic peptide (R = 0.4, *p* = 7.9e-12) and inversely correlated with left ventricular ejection fraction (R = −0.46, *p* = 9.8e-16).

**Conclusion:** We concluded that SSC5D was a specific response to HF. Serum SSC5D may function as a novel biomarker and therapeutic target for patients with HF.

## 1 Introduction

Heart failure (HF) is a systolic or diastolic dysfunction of the heart, which is the terminal stage of various cardiovascular diseases. Despite improvements in medication and management, the morbidity and mortality of HF remain high worldwide (Tedeschi et al., 2020). Currently, the clinical diagnosis of HF is mainly based on the patient’s symptoms, signs and left ventricular ejection fraction (LVEF) (Heidenreich et al., 2022).

Plasma B-type natriuretic peptide (BNP) and serum N-terminal pro-B-type natriuretic peptide (NT-proBNP) have been recommended as the gold standards for HF diagnosis in HF guidelines (Yancy et al., 2017). However, other non-cardiac diseases, such as renal failure (Tsutamoto et al., 2006), obesity (Das et al., 2005), *etc.*, can also change plasma BNP and serum NT-proBNP levels. Therefore, it is particularly important to find more biomarkers with higher specificity and sensitivity to improve the value of prediction and diagnosis of HF. In addition to classical HF biomarkers, studies on serum biomarkers of HF have increased. MicroRNAs are endogenous small non-coding RNAs that play a crucial role in cardiovascular diseases (Huang et al., 2018) and have been proven to be biomarkers for HF diagnosis and prognosis (Yang et al., 2021). Moreover, serum LL-37/cathelicidin-related antimicrobial peptide (CRAMP) (Zhou et al., 2020) and soluble ST2 receptor (Weinberg et al., 2003) have been shown to promote the diagnostic and prognostic value of HF. Given the deadly conditions of HF, the investigation of HF-specific responders and regulators will contribute to the diagnosis and treatment of clinical patients. In this study, we explored the secreted proteins of patients with HF and demonstrated that serum Soluble Scavenger with 5 Domains (SSC5D) was overly elevated in failing hearts compared to control group.

SSC5D is a member of the scavenger receptor cysteine-rich superfamily (SRCR-SF) (Gonçalves et al., 2009). Studies have demonstrated that numerous SRCR-SF members play essential roles in inflammation and immunity (Lee et al., 2019; Wang et al., 2021). Additionally, SRCR-SF has been reported to be associated with various cardiovascular diseases, such as collagen deposition, angiogenesis (Umana-Diaz et al., 2020), and atherosclerosis (Silva et al., 2016). However, the function of SSC5D in cardiovascular disease remains unknown. Therefore, this study investigates the diagnostic value of serum SSC5D for patients with HF.

## 2 Materials and methods

### 2.1 Patient’s characteristics

A total of 276 patients who were diagnosed with HF or control during hospitalization in Shanghai General Hospital between September 2020 and December 2021 were enrolled in this study. This study was approved by the Ethics Committee of Shanghai General Hospital (2018KY250). The study was performed under the Declaration of Helsinki and written informed consent was obtained from all patients.

HF is classified into the following categories based on LVEF: HFrEF (HF with reduced EF), LVEF≤40%; HFimpEF (HF with improved EF), previous LVEF≤40% and a follow-up measurement of LVEF>40%; HFmrEF (HF with mildly reduced EF), LVEF 41%–49%; HFpEF (HF with preserved EF), LVEF≥50% (Heidenreich et al., 2022). In this study, patients were grouped based on their symptoms and LVEF values: the HF group, LVEF<50% with typical symptoms of HF (Ponikowski et al., 2016), and the control group, LVEF≥50% without typical symptoms of HF. Patients who were diagnosed with acute infections, cancers, age<18 years, acute coronary syndrome, pregnancy, autoimmune diseases, or surgery within 1 month were prospectively excluded. Table 1 presents patient’s characteristics.

**TABLE 1:** Clinical characteristics of patients.

Characteristics	Controls (*N* = 148)	HF (*N =* 128)	*p*-Value
Age, years	61 (50–66)	61 (52–68)	0.4398
Male, n (%)	89 (60.1)	94 (73.4)	0.0197
Smoker, n (%)	24 (16.2)	23 (18.0)	0.6993
BMI, kg/m^2^	24.8 (22.8–27.1)	25.0 (22.9–27.3)	0.9217
SBP, mmHg	134 (120–146)	125 (113–137)	0.0004
DBP, mmHg	78 (70–84)	75 (68–83)	0.3011
Heart rate, bpm	78 (73–88)	82 (72–92)	0.2364
Medical history, n (%)			
Hypertension	67 (45.3)	64 (50.0)	0.4326
Diabetes mellitus	26 (17.6)	46 (35.9)	0.0005
Hypercholesterolemia	48 (32.4)	14 (10.9)	<0.0001
Atrial fibrillation	11 (7.4)	33 (25.8)	<0.0001
COPD	2 (1.4)	3 (2.3)	0.5376
Myocardial infarction	4 (2.7)	62 (48.4)	<0.0001
Anemia	0	5 (3.9)	0.0152
Treatment, n (%)			
ACE-I/ARB	59 (39.9)	118 (92.2)	<0.0001
Beta-blocker	49 (33.1)	109 (85.2)	<0.0001
Digoxin	1 (0.7)	17 (13.3)	<0.0001
Statin	94 (63.5)	100 (78.1)	0.0081
Antiplatelet/anticoagulant	71 (48.0)	76 (59.4)	0.0583
CCB	30 (20.3)	13 (10.2)	0.0209
Loop diuretic	1 (0.7)	71 (55.5)	<0.0001
Laboratory measurements			
NT-proBNP, pg/mL	48.6 (21.0–107.3)	1,147.0 (495.0–2,249.3)	<0.0001
HDL-c, mmol/L	1.1 (0.9–1.3)	1.0 (0.9–1.2)	0.0089
LDL-c, mmol/L	2.6 (2.1–3.2)	2.5 (1.9–3.2)	0.5810
eGFR, mL/min/1.73 m^2^	92.2 (79.6–99.3)	79.5 (67.3–94.5)	<0.0001
hs-CRP, mg/L	0.9 (0.3–1.9)	1.6 (0.8–5.0)	<0.0001
Sodium, mmol/L	142 (141–143)	141 (139–143)	0.0010
Creatinine, μmol/L	74 (65–84)	86 (71–100)	<0.0001
BUN, mmol/L	5.4 (4.5–6.5)	6.7 (5.1–8.1)	<0.0001
Cystatin C, mg/L	0.97 (0.85–1.08)	1.15 (1.03–1.33)	<0.0001
Hemoglobin, g/dL	138 (127–151.8)	140 (130–152)	0.6807
Total bilirubin, μmol/L	12.6 (9.9–16.8)	14.7 (10.7–20.4)	0.0063
Echocardiographic parameters			
LVEDd (mm)	48 (46–50)	63 (59–68)	<0.0001
LVESd (mm)	30 (28–31)	51 (46–57)	<0.0001
LAd (mm)	37 (35–40)	46 (43–51)	<0.0001
LVEF, %	68 (65–70)	37 (30–43.8)	<0.0001

Categorical variables are presented as n (percentage, %), and continuous variables are presented as median (interquartile range). *p*-value < 0.05 was considered statistically significant.

BMI, body mass index; SBP, systolic blood pressure; DBP, diastolic blood pressure; COPD, chronic obstructive pulmonary disease; ACE-I, angiotensin-converting enzyme inhibitors; ARB, Angiotensin II, receptor blocker; CCB, calcium channel blocker; NT-proBNP, N-terminal pro-B-type natriuretic peptide; HDL-c, high density lipoprotein cholesterol; LDL-c, low density lipoprotein cholesterol; eGFR, estimated glomerular filtration rate; hs-CRP, hypersensitive C-reactive protein; BUN, blood urea nitrogen; LVEDd, left ventricular end-diastolic dimension; LVESd, left ventricular end-systolic dimension; LAd, left atrial diameter; LVEF, left ventricular ejection fraction.

### 2.2 RNA-sequencing (RNA-seq) and single-cell RNA sequencing (scRNA-seq) data analysis

RNA-seq data of humans (Accession number: GSE165303, GSE46224, and GSE116250) and mice (Accession number: GSE95755) were downloaded from Gene Expression Omnibus (https://www.ncbi.nlm.nih.gov/geo/). Raw data were transformed into fragments per kilobase of exon model per million mapped fragments (FPKM), reads per kilobase per million mapped reads (RPKM), and counts per million (CPM) using the R statistical software for further analysis. The expression pattern of *Ssc5d* was derived from previously published scRNA-seq data (Zhuang et al., 2020). The human cardiac single nucleus RNA sequencing (snRNA-seq) transcriptomics data from the Kuppe et al. study was downloaded from: https://cellxgene.cziscience.com/collections/8191c283-0816-424b-9b61-c3e1d6258a77) (Kuppe et al., 2022). To re-analyze the scRNA-seq data, we performed a quality control of single cell by choosing individual live cell among each dataset. In brief, both the count of features and mitochondria were defined as the cut-offs. Then the data from all individual cell was screened based on the original article criteria prior to further analysis. When the quality control was completed, we normalized each cell characteristic by dividing or multiplying all unique molecular identifiers by 10,000 to get the value in per million transcripts, and then performed logarithmic transformation using RStudio.

### 2.3 Secreted proteins data analysis

The secreted proteins data were downloaded from the IUPHAR/BPS Guide to Pharmacology website (https://www.guidetopharmacology.org/).

### 2.4 Myocardial infarction (MI) and transverse aortic constriction (TAC) model

Male C57BL/6 mice (6–8 weeks old) were purchased from Sipper-BK Laboratory Animal Co., Ltd (Shanghai, China). All animal experimental procedures were approved by the Animal Welfare and Ethics Committee of Shanghai General Hospital (2021AW035) and conducted in accordance with the National Institutes of Health (NIH) Guide for the Care and Use of Laboratory Animals. TAC and MI surgery were performed based on the previous literature to construct failing heart model (n = 5–7) (Du et al., 2020; Zhuang et al., 2022).

### 2.5 RNA extraction and real-time quantitative PCR (RT-qPCR) assays

Heart tissues were collected from mice after MI (14 and 28 days) or TAC (1 and 4 weeks) surgery and the sham group. Total RNA was extracted using RNAiso Plus (Takara, Japan) and cDNA was amplified using the PrimeScript™ RT Master Mix kit (Takara, Japan) according to the manufacturer’s protocol. Quantitative PCR analysis was performed using the TB Green^®^ Premix Ex Taq™ kit (Takara, Japan) and the QuantStudio™ 7 Flex Real-Time PCR System (Applied Biosystems Co., United States). The specific qPCR primer sequences were as follows (5′-3′):

Mouse *Ssc5d* Forward primer: GCG​TCG​TCT​GTG​TAG​GTC​AG; Mouse Ssc5d Reverse primer: AGC​GTG​AGT​TAT​AGG​GGG​CT; Mouse Gapdh Forward primer: CCG​CAT​CTT​CTT​GTG​CAG​T; Mouse Gapdh Reverse primer: CAT​CAC​CTG​GCC​TAC​AGG​AT; Gapdh was used as an endogenous control and the 2^^−ΔΔCT^ method was used to analyze the data.

### 2.6 Blood samples collection and ELISA assays

Blood samples were collected from patients with HF and the control group, centrifuged at 3,000 rpm for 20 min to obtain serum, and stored at −80°C for further ELISA assays. Serum SSC5D concentrations were measured using the Human SSC5D/Soluble scavenger receptor cysteine-rich domain-containing protein SSC5D ELISA Kit (catalog no: #EK21098, SAB, United States), according to the manufacturer’s instructions. Human serum samples were diluted 1:100 in sample diluent. All serum samples were measured by a researcher who was oblivious to the patient’s clinical data.

### 2.7 Statistical analysis

IBM SPSS Statistics (version 26, 2019) and R statistical software (version 4.0.4, 2021) were used to analyze data. The experimental data were represented as mean ± SEM. Two-tailed unpaired Student’s t-test was performed to compare the difference between 2 groups. One-way ANOVA followed by the Tukey *post hoc* test was used to evaluate the difference between ≥3 groups. Categorical variables were presented as numbers (percentages, %) and were compared using the chi-square test. The Kolmogorov-Smirnov test was used to assess the normality of the continuous variables. If the continuous variables did not conform to the normality distribution, they were presented as median (interquartile range) and compared with the Mann-Whitney *U* test. The SSC5D concentration values were transformed into a normal distribution using a logarithm of 10 and divided into quartiles for further analysis. Spearman’s rank correlation coefficients were used to evaluate the relationships between serum SSC5D levels and NT-proBNP, LVEF, estimated glomerular filtration rate (eGFR), blood urea nitrogen (BUN), cystatin c, and creatinine. A logistic regression model was performed to evaluate the association between HF and risk factors of HF. Sex, age, body mass index (BMI), diabetes mellitus, hypertension, hemoglobin, creatinine, low-density lipoprotein cholesterol (HDL-c), hypersensitive C-reactive protein (hs-CRP), BUN, Haemoglobin A1c (HbA1c), and eGFR were adjusted. The receiver operating characteristic (ROC) curve was used to assess the diagnostic value and determine the optimal cut-off value with or without SSC5D. The two-sided *p*-value < 0.05 was considered statistically significant.

## 3 Results

### 3.1 *SSC5D* transcript is elevated in failing heart

**FIGURE 1:**
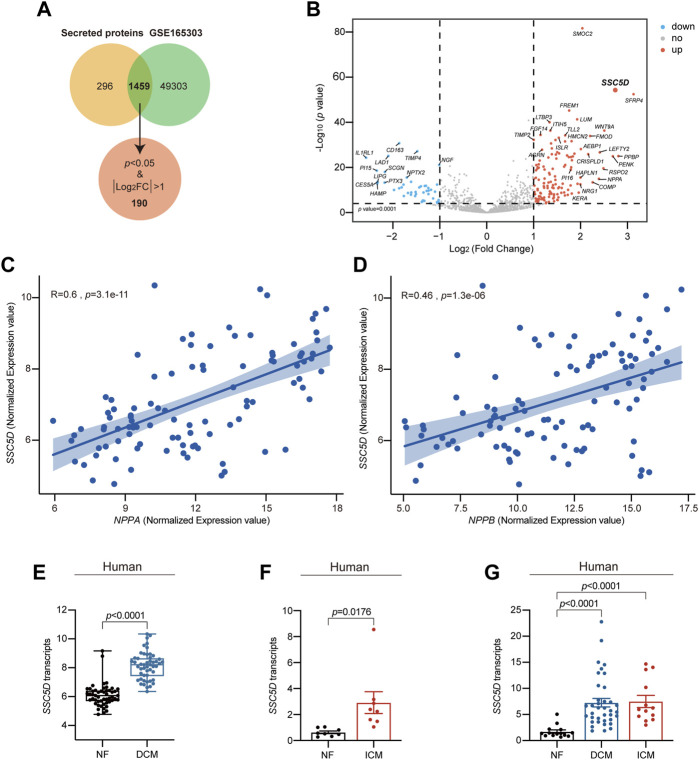
*SSC5D* transcript is highly elevated in failing hearts. **(A)** The Venn diagram shows the common genes between the secreted proteins and GSE165303 data. **(B)** The volcano map shows the significantly differentially expressed genes among 1,459 common genes. Red dots, significantly elevated genes; Blue dots, significantly downregulated genes; Grey dots, non-significantly altered genes. **(C, D)** The correlation between *SSC5D* (log2 [normalized counts]) and *NPPA*, *NPPB* (log2 [normalized counts]) in 51 NF and 50 DCM hearts RNA-seq data (GSE165303). **(E)** Normalized *SSC5D* expression values (log2 [normalized counts]) in 51 NF and 50 DCM human hearts RNA-seq data (GSE165303). **(F)** Normalized *SSC5D* expression (RPKM) in 8 NF and 8 ICM human hearts RNA-seq data (GSE46224). **(G)** Normalized *SSC5D* expression (RPKM) in 14 NF, 13 ICM and 37 DCM human hearts RNA-seq data (GSE116250). NF, non-failing; DCM, dilated cardiomyopathy; ICM, ischemic cardiomyopathy; *p*-value < 0.05 was considered statistically significant.

To investigate the role of secreted proteins in cardiovascular diseases, we first collected a list of secreted proteins data. Subsequently, we re-analyzed their expression patterns in failing and non-failing (NF) heart tissue RNA-seq (GSE165303) data (Feng et al., 2022). We identified 1,459 genes in common (Figure 1A). Furthermore, by setting *p* < 0.05 and |log_2_FoldChange|>1, we identified 190 secreted proteins that were differentially expressed in the hearts of patients with HF, and *SSC5D* was one of the most differentially upregulated genes (Figure 1B). Furthermore, through Pearson correlation analysis in R, we discovered a significant positive correlation between *SSC5D* and the cardiac hypertrophy marker genes A-type natriuretic peptide (*NPPA*) (R = 0.6, *p* = 3.1e-11) and B-type natriuretic peptide (*NPPB*) (R = 0.46, *p* = 1.3e-6) (Figures 1C, D). To verify the results of increased *SSC5D* expression, a series of human failing heart tissues (GSE165303, GSE46224, and GSE116250) RNA-seq data were re-analyzed to compare the expression of *SSC5D*. From RNA-seq data analysis, we found that *SSC5D* expression was significantly upregulated in the failing group (dilated cardiomyopathy, DCM; ischemic cardiomyopathy, ICM) compared to the NF group (Figures 1E–G).

**FIGURE 2:**
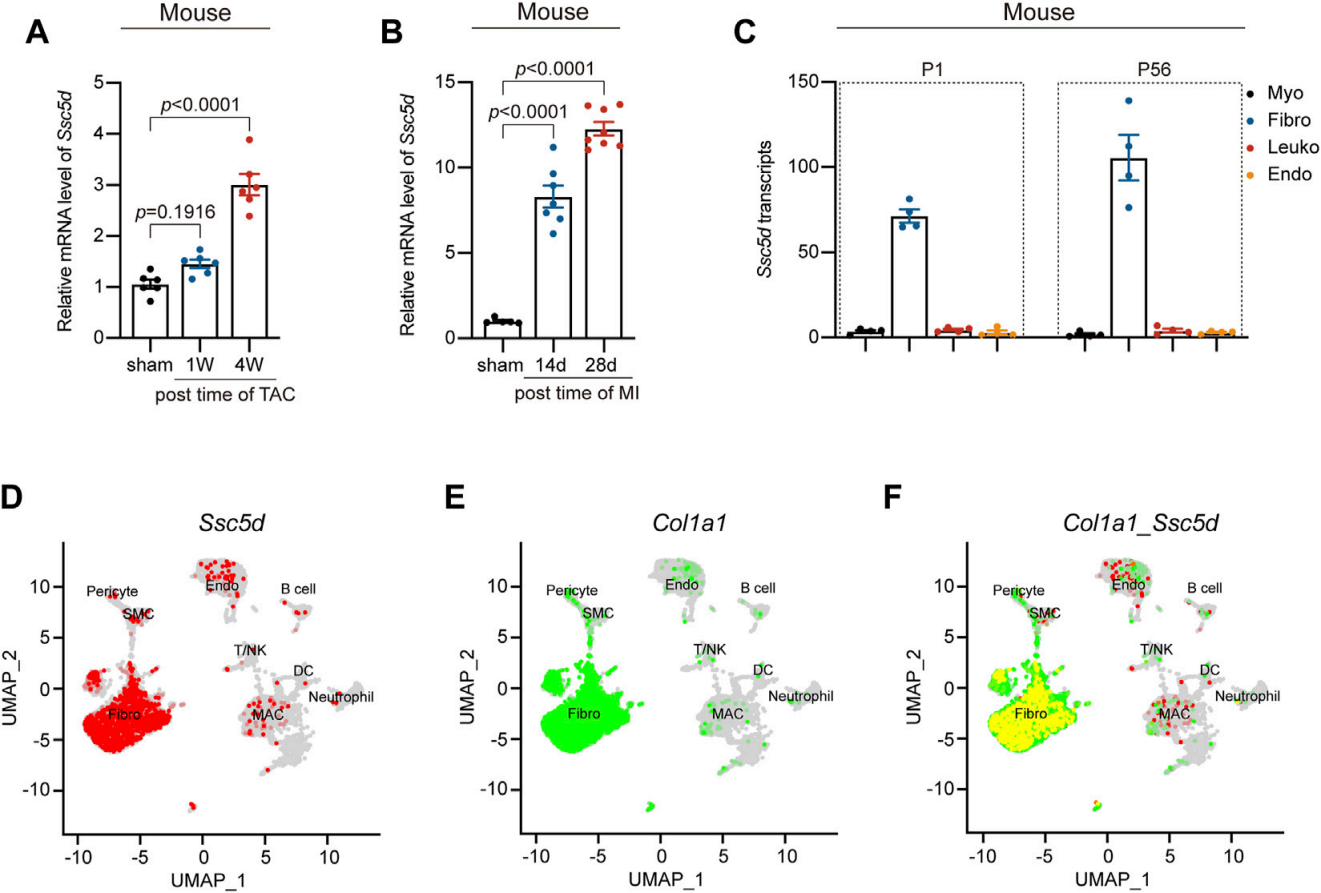
*Ssc5d* mRNA levels were significantly elevated in MI and TAC mouse models. **(A,B)** The relative mRNA expression of *Ssc5d* after TAC (1 and 4 W) (n = 6) and MI (14 and 28 days) (n = 5–7) surgery. **(C)** Normalized *Ssc5d* expression (CPM) in each cell type at neonatal (P1) or adult stages (P56) mouse hearts (GSE95755). Myo (black), cardiomyocytes; Fibro (blue), fibroblasts; Leuko (red), leukocytes; Endo (orange), endothelial cells. **(D–F)** UMAP plot of *Ssc5d* and *Col1a1* co-expression in sham mice cardiac non-cardiomyocyte clusters. Red, *Ssc5d*; Green, *Col1a1*; Yellow, *Ssc5d* and *Col1a1* co-expression. Fibro, fibroblasts; SMC, smooth muscle cell; MAC, macrophage; DC, dendritic cell; Endo, endothelial; CPM, counts per million; W, week; d, day. *p*-value < 0.05 was considered statistically significant.

To further verify the human failing heart RNA-seq data, we constructed mouse TAC and MI models and collected cardiac tissue at different times after surgery. We found that the *Ssc5d* mRNA expression was significantly elevated at 4 weeks (approximately 2-fold, *p* < 0.0001) after TAC surgery compared to that in the sham group (Figure 2A). Similarly, compared with the sham group, *Ssc5d* mRNA levels were significantly elevated at 14 and 28 days after MI surgery, and the highest fold increase (approximately 11-fold, *p* < 0.0001) was observed at day 28 (Figure 2B). To further determine the origin of *Ssc5d*, we re-analyzed mouse heart RNA-seq data (GSE95755) (Quaife-Ryan et al., 2017), which sorted cardiac cardiomyocytes, fibroblasts, leukocytes, and vascular endothelial cells for RNA-seq analysis. We discovered that *Ssc5d* levels were significantly higher in fibroblasts than in other cell populations in both neonatal (P1) and adult (P56) mouse hearts (Figure 2C). Additionally, Zhuang et al. have integrated three representative mouse heart scRNA-seq datasets, including 27,349 non-cardiomyocytes (macrophages, fibroblasts, endothelia, and lymphocytes) isolated from myocardial infarction or sham heart tissue (Zhuang et al., 2020). We contacted the authors and re-analyzed their data to explore the expression profile of *Ssc5d* in non-cardiomyocytes and revealed that *Ssc5d* was markedly co-expressed with *Col1a1*-positive fibroblasts. Moreover, we re-analyzed the previous published human cardiac single-nucleus RNA sequencing data (snRNA-seq) (Kuppe et al., 2022). By analyzing this snRNA-seq data, we showed that *SSC5D* was expressed predominantly in fibroblasts, to the less extent in endothelial cells or myeloid cells (Supplementary Figures S1A, B). This is consistent with our previous findings that SSC5D is mainly expressed in fibroblasts. Although it is possible that circulating Ssc5d is synthesized by other cells, our results demonstrated that it is mainly present in fibroblasts in the heart. Taken together, these results suggest that cardiac Ssc5d was mainly derived from fibroblasts and was markedly upregulated after MI and TAC surgery.

### 3.2 Patient’s baseline characteristics

To further clarify the relationship between serum SSC5D and HF, we collected 276 blood samples and measured serum SSC5D concentrations in all patients using an ELISA Kit. The 276 enrolled patients were grouped into two based on their symptoms and LVEF values. Table 1 presents the baseline data of the patients. There were no significant differences in age, smoking status, BMI, diastolic blood pressure (DBP), and heart rate between the control and HF groups. More men were included in the HF group than in the control group and more HF patients have a medical history of diabetes mellitus, anemia, atrial fibrillation, and myocardial infarction. The number of patients treated with angiotensin-converting enzyme inhibitors/angiotensin II receptor blockers (ACE-I/ARB), beta-blockers, digoxin, statins, calcium channel blockers (CCB), and loop diuretic drugs was significantly higher in the HF group than in the control group. However, no significant difference was observed in patients receiving antiplatelet or anticoagulant drug treatment. Compared with the control group, blood creatinine, total bilirubin, NT-proBNP, hs-CRP, and BUN levels were higher in the HF group, whereas eGFR, high-density lipoprotein cholesterol (HDL-c), and sodium levels were slightly lower in the HF group. Furthermore, the echocardiographic results indicated a significant decrease in LVEF but an increased left ventricular end-diastolic dimension (LVEDd), left ventricular end-systolic dimension (LVESd), and left atrial diameter (LAd) in the HF group compared with the control group.

### 3.3 Association of serum SSC5D with risk factors of HF

**FIGURE 3:**
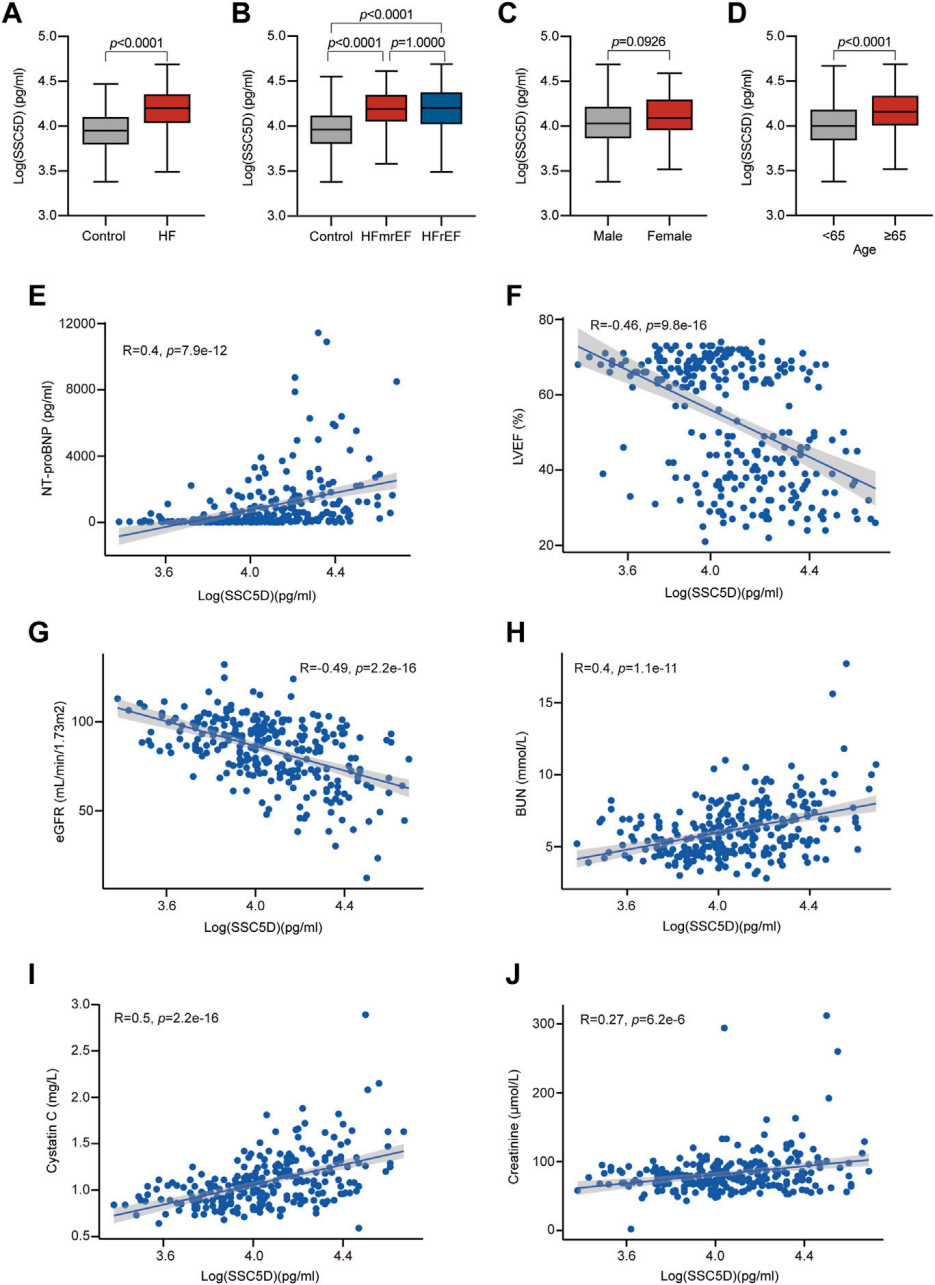
Serum SSC5D levels increased in HF patients. **(A)** Serum SSC5D levels in HF patients compared with control. **(B)** Serum SSC5D levels in control, HFmrEF, and HFrEF subjects. **(C, D)** Serum SSC5D levels in male and female, <65 and ≥65 years old subjects, respectively. **(E–J)** The correlation between serum SSC5D levels and NT-proBNP, LVEF, eGFR, BUN, cystatin c, and creatinine. The SSC5D concentrations were transformed by a logarithm of 10 to obtain normality. *p*-value < 0.05 was considered statistically significant.

We discovered that the SSC5D levels were significantly elevated in the HF group compared with the control group [15,789.35 (10,745.32–23110.65) pg/mL, 95% CI (16,263.01–19655.43) vs. 8,938.72 (6,154.97–12778.81) pg/mL, 95% CI (9,337.50–11142.93); *p* < 0.0001] (Figure 3A). Furthermore, patients with HF were grouped into two, HFmrEF (n = 37) and HFrEF (n = 86) groups, and we discovered that the serum SSC5D levels in the HFmrEF and HFrEF groups were significantly higher than those in the control group (Figure 3B). However, no statistical difference was observed between the HFmrEF and HFrEF groups (Figure 3B).

We then analyzed the relationship between serum SSC5D levels and clinical HF risk factors. We discovered that serum SSC5D levels were higher in patients aged ≥65 years than those aged <65 years (Figure 3D). Patients with a history of hypertension and diabetes had higher serum SSC5D levels (Table 2). Moreover, elevated NT-proBNP and decreased LVEF were highly correlated with higher serum SSC5D levels (R = 0.4, *p* = 7.9e-12, and R = −0.46, *p* = 9.8e-16, respectively) (Figures 3E, F). Serum SSC5D levels were significantly correlated with indicators of renal dysfunction, such as eGFR, BUN, cystatin c, and creatinine (Figures 3G–J). However, no statistical difference in serum SSC5D levels was observed between the sexes and smoking status (Figure 3C; Table 2).

**TABLE 2:** Correlation analysis between HF risk factors and SSC5D concentrations.

Variables	SSC5D (pg/mL)	*p*-Value
Gender		
Male (n = 183)	10,816.41 (7,292.48–16,740.40)	0.0956
Female (n = 93)	12,256.01 (8,949.68–20,167.58)	
Age		
<65 (n = 173)	10,102.50 (6,889.57–15,352.32)	<0.0001
≥65 (n = 103)	14,575.00 (9,912.62–21,872.13)	
Smoking		
Yes (n = 47)	10,696.58 (6,799.49–14,196.77)	0.2587
No (n = 229)	11,543.20 (7,876.62–18,328.31)	
Hypertension		
Yes (n = 131)	14,120.30 (10,053.31–21,035.89)	<0.0001
No (n = 145)	9,561.05 (6,459.68–14,248.90)	
Hypercholesterolemia		
Yes (n = 62)	9,764.05 (7,331.49–13389.56)	0.0017
No (n = 214)	12,113.56 (7,892.27–19,868.04)	
Diabetes		
Yes (n = 72)	15,790.19 (11,298.94–23,199.95)	<0.0001
No (n = 204)	10,075.91 (6,977.58–15,562.41)	
LVEF (*R* = -0.46, *p* = 9.8e-16)		
≥50% (n = 153)	9,117.39 (6,294.03–13,132.71)	
41%–49% (n = 37)	15,507.25 (11,187.34–22,366.36)	*p* ^*1*^ < 0.0001
≤40% (n = 86)	15,789.35 (10,373.27–23,843.91)	*p* ^*2*^ < 0.0001
NT-proBNP (*R* = 0.4, *p* = 7.9e-12)		
<125 pg/mL (n = 116)	8,488.70 (5,824.16–12,065.34)	<0.0001
≥125 pg/mL (n = 156)	14,813.76 (10,102.50–21,660.74)	

Data are shown as median (interquartile range) and used Mann-Whitney *U* test between two groups. *p*-value < 0.05 was considered statistically significant.

*p* ^1^ and *p* ^*2*^ were the results of comparison between LVEF≥50% group with LVEF, 41%–49% and≤40% group in serum SSC5D levels, respectively.

Pearson correlation coefficient in R was used to analyze the correlation between serum SSC5D levels with the continuous LVEF, and NT-proBNP, level.

### 3.4 Predictive value of serum SSC5D

These results clarified that serum SSC5D levels were significantly elevated in patients with HF. Further, we explored the diagnostic value of serum SSC5D in patients with HF. Through univariate and multivariate binary logistic regression models analysis, we discovered that log-transformed serum SSC5D levels were strongly positively associated with the prevalence of HF (OR:3.23, 95% CI:2.32–4.50, *p* < 0.001) (Table 3). Meanwhile, we divided the log-transformed SSC5D into tertiles and discovered that the highest SSC5D tertile was associated with a higher risk of HF (OR:11.02, 95% CI:5.53–21.97, *p* < 0.001). Subsequently, we adjusted the covariates to analyze the correlation between the SSC5D score and HF. After adjusting age and sex in Model 1 and other covariates (sex, age, BMI, diabetes mellitus, hypercholesterolemia, hypertension, hemoglobin, creatinine, LDL-c, hs-CRP, BUN, HbA1c, and eGFR) in Model 2, log-transformed serum SSC5D levels were highly positively associated with a higher risk of HF (OR:3.60, 95% CI:2.53–5.11, *p* < 0.001 and OR:3.40, 95% CI:2.10–5.51, *p* < 0.001, respectively) (Table 3). Additionally, the highest SSC5D tertile had a higher risk of HF in Models 1 and 2 (OR:17.70, 95% CI:7.78–40.24, *p* < 0.001 and OR:11.83, 95% CI:4.30–33.09, *p* < 0.001, respectively) (Table 3).

**TABLE 3:** Serum SSC5D levels were highly associated with the risk factors of HF.

	Unadjusted	*p*-Value	Model 1	*p*-Value	Model 2	*p*-Value
	OR (95% CI)		OR (95% CI)		OR (95% CI)	
logSSC5D (per SD)	3.23 (2.32–4.50)	<0.001	3.60 (2.53–5.11)	<0.001	3.40 (2.10–5.51)	<0.001
SSC5D tertiles	3.31 (2.35–4.66)	<0.001	4.16 (2.77–6.26)	<0.001	3.41 (2.05–5.67)	<0.001
Tertile 1	1 (referent)		1 (referent)		1 (referent)	
Tertile 2	3.61 (1.87–6.97)	<0.001	4.90 (2.38–10.10)	<0.001	4.03 (1.71–9.48)	0.001
Tertile 3	11.02 (5.53–21.97)	<0.001	17.70 (7.78–40.24)	<0.001	11.83 (4.30–33.09)	<0.001

SSC5D concentration was normalized by log_10_ transformation to obtain normality and divided into tertiles.

Model 1: Adjusted for age and sex.

Model 2: Adjusted for sex, age, BMI, diabetes mellitus, hypercholesterolemia, hypertension, hemoglobin, creatinine, low-density lipoprotein cholesterol, hypersensitive C-reactive protein, blood urea nitrogen, HbA1c, and estimated glomerular filtration rate.

**FIGURE 4:**
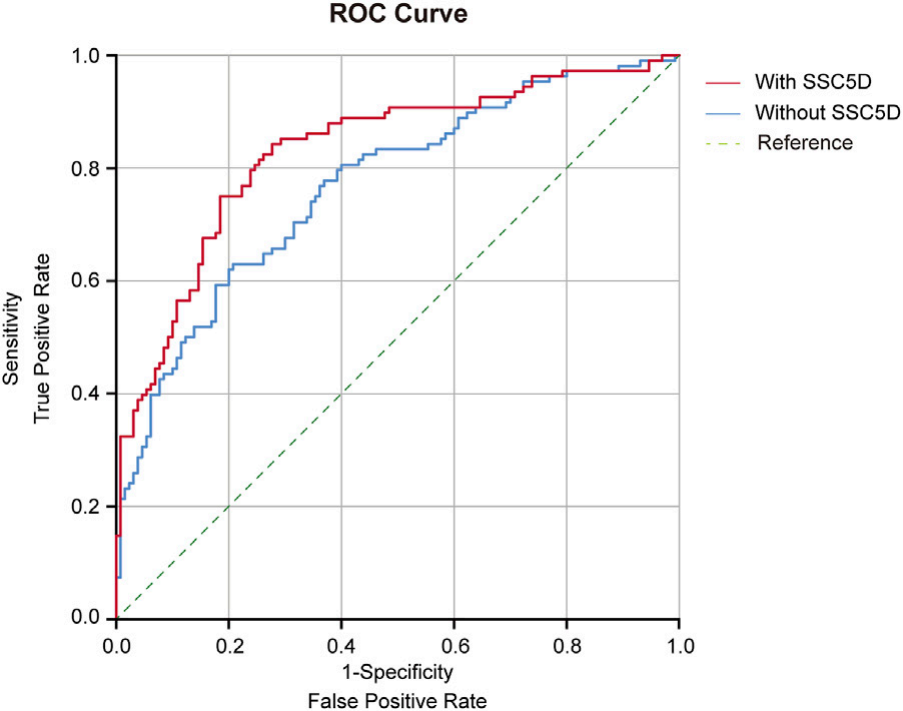
Receiver operating characteristic (ROC) curve with area under the curve (AUC) for Model 2 with SSC5D or without SSC5D. Red curve: ROC curve for Model 2 with SSC5D; Blue curve: ROC curve for Model 2 without SSC5D; Green curve: Reference. Model 2: sex, age, BMI, diabetes mellitus, hypercholesterolemia, hypertension, hemoglobin, creatinine, LDL-c, hs-CRP, BUN, HbA1c, and eGFR.

Receiver operating characteristic (ROC) curves were generated to investigate the diagnostic accuracy of the SSC5D for HF. First, we determined the diagnostic accuracy of individual SSC5D value on heart failure, and the ROC curve results showed that the sensitivity and specificity value was 0.750 and 0.676, respectively, and the AUC value was 0.773 (Supplementary Figure S2). In addition, we discovered that the area under the curve (AUC) value of SSC5D was 0.831, which was markedly improved compared to that without SSC5D (AUC: 0.768) in Model 2 (Figure 4; Table 4). The ROC curve analysis showed that the specificity and sensitivity were 0.723 and 0.843, respectively, and the optimal cut-off value of SSC5D concentration for predicting HF was 10,853.98 pg/mL in Model 2 (Figure 4; Table 4). Taken together, these results indicate that SSC5D is a sensitive indicator of HF and may serve as a therapeutic target for treating HF.

**TABLE 4:** ROC curve analysis for SSC5D to predict the diagnosis of HF.

	ROC aera (AUC)	95% CI	Specificity	Sensitivity
Without SSC5D	0.768	0.708–0.828	0.792	0.630
With SSC5D	0.831	0.777–0.884	0.723	0.843

## 4 Discussion

In this study, we identified a sensitive indicator of HF by screening for the expression of secreted genes in a human RNA-seq dataset. The transcriptional level of *SSC5D* was significantly higher in the failing heart than in the non-failing heart in both clinical and pre-clinical models. Furthermore, we demonstrated that the *Ssc5d* mRNA levels were markedly elevated after TAC and MI surgery. Combining the scRNA-seq data and published RNA-seq data, we demonstrated that *Ssc5d* is predominantly expressed in cardiac fibroblasts in the mouse heart. Notably, the measurement of serum SSC5D concentrations revealed that patients with HF had higher SSC5D levels than those in the control group, which may serve as a therapeutic target for treating HF.

SSC5D is a new soluble protein that has been identified as a new family member of glycoproteins of the scavenger receptor cysteine-rich superfamily. A previous study indicated that SSC5D is predominantly expressed in monocytes/macrophages and T lymphocytes (Gonçalves et al., 2009).

However, our results demonstrated that *Ssc5d* was predominantly expressed in cardiac fibroblasts but was rarely expressed in cardiomyocytes, leukocytes, and endothelial cells. We speculate that this may be attributed to differential expression patterns in various tissues. However, whether *Ssc5d* is derived from cardiac fibroblasts must be verified in the future. Furthermore, studies have shown that abnormal expression of SSC5D is associated with various diseases, such as multiple myeloma (Laï et al., 1995), primary megakaryoblastic leukemia, and acute myeloid leukemia (Alvarez et al., 2001). Additionally, SSC5D is associated with inflammation. The protein level of SSC5D is significantly increased in the synovial fluid of patients with osteoarthritis (Balakrishnan et al., 2014). To date, no reports on the correlation between serum SSC5D levels and the incidence of HF exist. By re-analyzing the RNA-seq data of HF, this study demonstrated for the first time that fibroblast-derived SSC5D was remarkably upregulated in failing conditions.

Similar to SSC5D, S4D-SRCRB is a soluble member of group B SRCR-SF, which is considered to resemble SSC5D in amino acid composition and domain organization (Gonçalves et al., 2009). S4D-SRCRB plays a crucial role in innate immunity (Padilla et al., 2002). CD5L is also one of the SRCR-SF domains and contains three SRCR domains. The elevated CD5L levels often occur in infectious and inflammatory processes (Sanchez-Moral et al., 2021). Agra-Bermejo et al. suggested that isoproterenol treatment of patients with HF or atrial fibrillation significantly increased CD5L secretion from epicardial adipose tissue, which may activate the toll-like receptor 4/nuclear factor-kappa B (NF-κB) signaling pathway and produce pro-inflammatory cytokines (Agra-Bermejo et al., 2020). Circulating CD5L is highly correlated with the risk of cardiovascular events in patients with chronic kidney dysfunction (Castelblanco et al., 2021). Moreover, CD5L promotes atherosclerosis by increasing the formation of macrophage foam cells and CD36-mediated oxidized low-density lipoprotein uptake (Amézaga et al., 2014). Similarly, Scavenger Receptors Stabilin-1 and Stabilin-2 were associated with atherosclerosis, and inhibition of Stabilin-1/Stabilin-2 can significantly reduce Erg1 expression in mononuclear macrophages, thus reducing the progression of atherosclerosis (Manta et al., 2022). Lysyl Oxidase-like2 (LOXL2), one of SRCR-SF, plays a key role in cardiovascular diseases, and directedly interacted with collagen IV and fibronectin to regulate deposition of extracellular matrix (ECM) components (Umana-Diaz et al., 2020). Additionally, LOXL2 regulates the PI3K/AKT/mTORC1 signaling pathway to stimulate myofibroblast transformation in cardiac fibroblasts (Yang et al., 2016). Thus, we hypothesized that SSC5D may contribute to the augmentation of the cardiac immune response and ECM deposition *via* NF-κB and PI3K/AKT/mTORC1 or other pathways, further exacerbating cardiac dysfunction, which should be validated in the future.

## 5 Limitations

This study has some limitations: 1) Although we discovered that serum SSC5D levels are significantly increased in patients with HF, the role of SSC5D in HF remains unknown. 2) Although our findings suggest that SSC5D is mainly expressed in cardiac fibroblasts, it remains unclear whether SSC5D is associated with fibrosis during cardiac remodeling after HF and the underlying pathways. Therefore, further studies are required to elucidate the function and mechanisms of SSC5D in HF progression. 3) We did not validate the role of serum SSC5D in heart failure with a new validation cohort. Therefore, in future studies, a bigger sample size would be used to clarify the diagnostic and prognostic values of SSC5D on clinical heart failure patients.

## 6 Conclusion

In conclusion, this study demonstrates that elevated serum SSC5D levels are markedly associated with HF and function as a novel biomarker of clinical HF. Targeting SSC5D may provide therapeutic benefits for patients with HF in the future.

## Data Availability

The datasets presented in this study can be found in online repositories. The names of the repository/repositories and accession number(s) can be found in the article/Supplementary Material.
