# Supplementary material for: The serum soluble scavenger with 5 domains levels: A novel biomarker for individuals with heart failure
Source: Front Physiol. 2023 Apr 13;14:1140856. doi: 10.3389/fphys.2023.1140856 (PMC10133869; doi:10.3389/fphys.2023.1140856)
Supplement: Supplementary file 1 [file DataSheet1.PDF]

Supplementary Figure 1

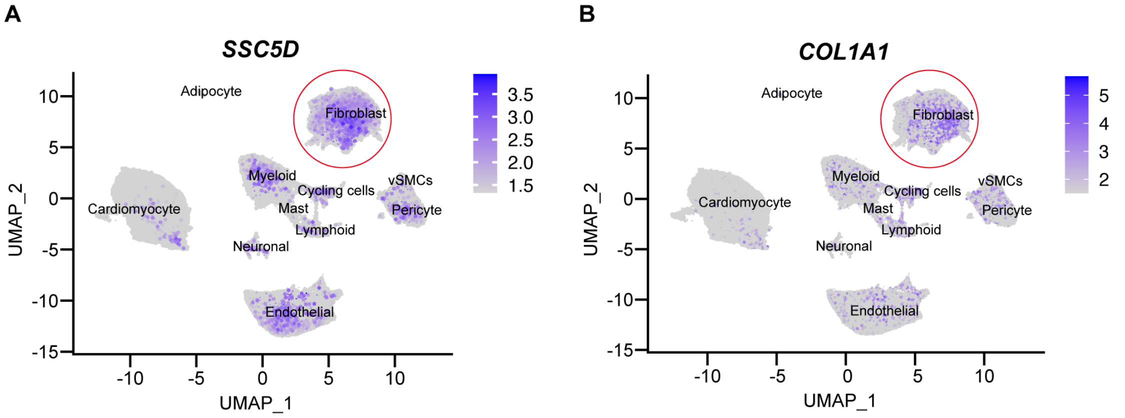

Supplementary Figure 1. (A and B) UMAP plot of the co-expression of *SSC5D* and *COL1A1* from the snRNA-seq data of all 31 human heart samples in the cardiac cell clusters.

Supplementary Figure 2

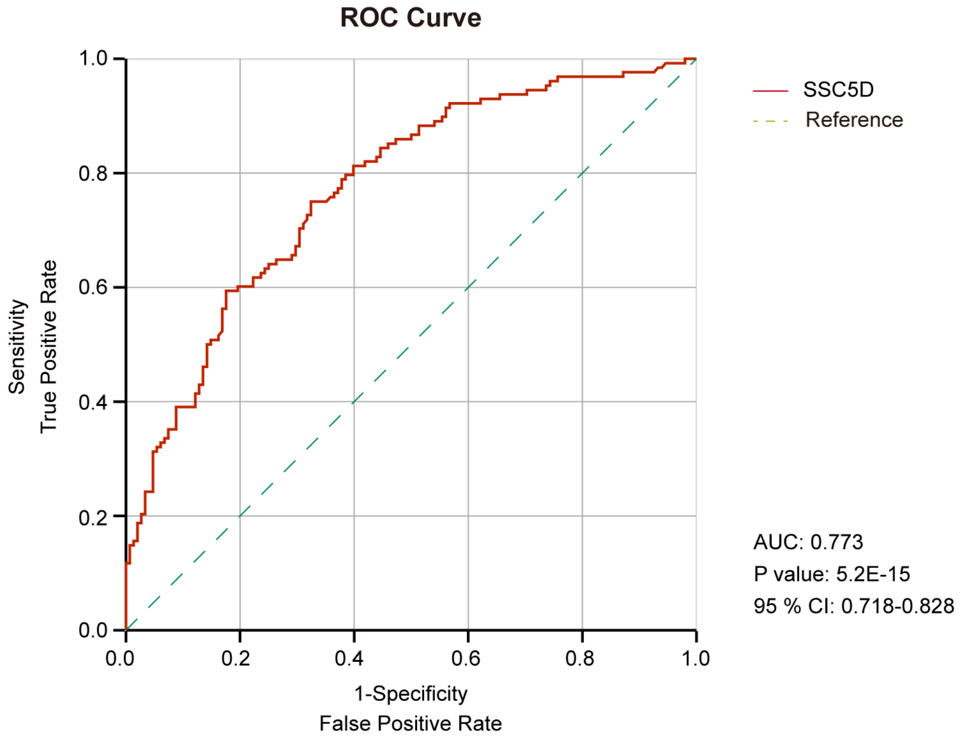

Supplementary Figure 2. Receiver operating characteristic (ROC) curve with area under the curve (AUC) of SSC5D for the diagnosis of heart failure. Red curve: ROC curve of SSC5D; Green curve: Reference. The SSC5D concentrations were transformed by a logarithm of 10 to obtain normality.
